# The Triglyceride-Glucose Index, an Insulin Resistance Marker, Was Non-linear Associated With All-Cause and Cardiovascular Mortality in the General Population

**DOI:** 10.3389/fcvm.2020.628109

**Published:** 2021-01-14

**Authors:** Xiao-cong Liu, Guo-dong He, Kenneth Lo, Yu-qing Huang, Ying-qing Feng

**Affiliations:** ^1^Department of Cardiology, Guangdong Cardiovascular Institute, Guangdong Provincial People's Hospital, Guangdong Academy of Medical Sciences, Guangzhou, China; ^2^Department of Epidemiology, Center for Global Cardio-Metabolic Health, Brown University, Providence, RI, United States; ^3^Department of Applied Biology and Chemical Technology, The Hong Kong Polytechnic University, Hung Hom, China

**Keywords:** insulin resistance, all-cause mortality, cardiovascular mortality, NHANES, risk factors, triglyceride-glucose index

## Abstract

**Background:** The triglyceride-glucose (TyG) index could serve as a convenient substitute of insulin resistance (IR), but epidemiological evidence on its relationship with the long-term risk of mortality is limited.

**Methods:** Participants from the National Health and Nutrition Examination Survey during 1999–2014 were grouped according to TyG index (<8, 8–9, 9–10, >10). Cox regression was conducted to compute the hazard ratios (HRs) and 95% confidence interval (CI). Restricted cubic spline and piecewise linear regression were performed to detect the shape of the relationship between TyG index and mortality.

**Results:** A total of 19,420 participants (48.9% men) were included. On average, participants were followed-up for 98.2 months, and 2,238 (11.5%) and 445 (2.3%) cases of mortality due to all-cause or cardiovascular disease were observed. After adjusting for confounders, TyG index was independently associated with an elevated risk of all-cause (HR, 1.10; 95% CI, 1.00–1.20) and cardiovascular death (HR, 1.29; 95% CI, 1.05–1.57). Spline analyses showed that the relationship of TyG index with mortality was non-linear (All non-linear *P* < 0.001), and the threshold value were 9.36 for all-cause and 9.52 for cardiovascular death, respectively. The HRs above the threshold point were 1.50 (95% CI, 1.29–1.75) and 2.35 (95% CI, 1.73–3.19) for all-cause and cardiovascular death. No significant difference was found below the threshold points (All *P* > 0.05).

**Conclusion:** Elevated TyG index reflected a more severe IR and was associated with mortality due to all-cause and cardiovascular disease in a non-linear manner.

## Introduction

Insulin resistance (IR) is an insensitivity state of the peripheral tissues and has been recognized as a major pathological feature of type 2 diabetes (1, 2). IR implies the defects in glucose uptake, reduction in glycogenesis synthesis, and decrement in suppressing lipid oxidation (3). In such a condition, to maintain glucose homeostasis, insulin secretion is increased and leads to chronic hyperinsulinemia, and therefore elevated oxidative stress and inflammatory responses (1, 4). IR may also impair lipid and serum urate metabolism, and therefore associated with metabolic syndrome (5, 6). Thus, quantifying IR quickly and accurately is important for clinical practice. The most established method to measure IR is euglycemic–hyperinsulinemic clamp. However, this method has limited applicability due to time and monetary cost (7). Another approach for measurement refers to Homeostatic Model Assessment for Insulin Resistance (HOMA-IR), but is also expensive due to insulin measurement (8).

Previous studies have demonstrated that lipotoxicity and glucotoxicity are key factors in IR modulation (1, 3). The triglyceride-glucose (TyG) index is the logarithmized product of fasting triglycerides and fasting glucose and has been proposed as the alternative indicator of IR due to its relevance to lipotoxicity and glucotoxicity (9, 10). TyG index has demonstrated a close relationship with cardiometabolic outcomes, namely diabetes, arterial stiffness, hypertension, cardiovascular disease (CVD), stroke and obesity-related cancers in previous studies (11–18). However, epidemiological evidence on its relationship with all-cause mortality and CVD death is limited. Therefore, we will address the knowledge gap in the United States (US) general population.

## Materials and Methods

### Study Design and Participants

**Figure 1:**
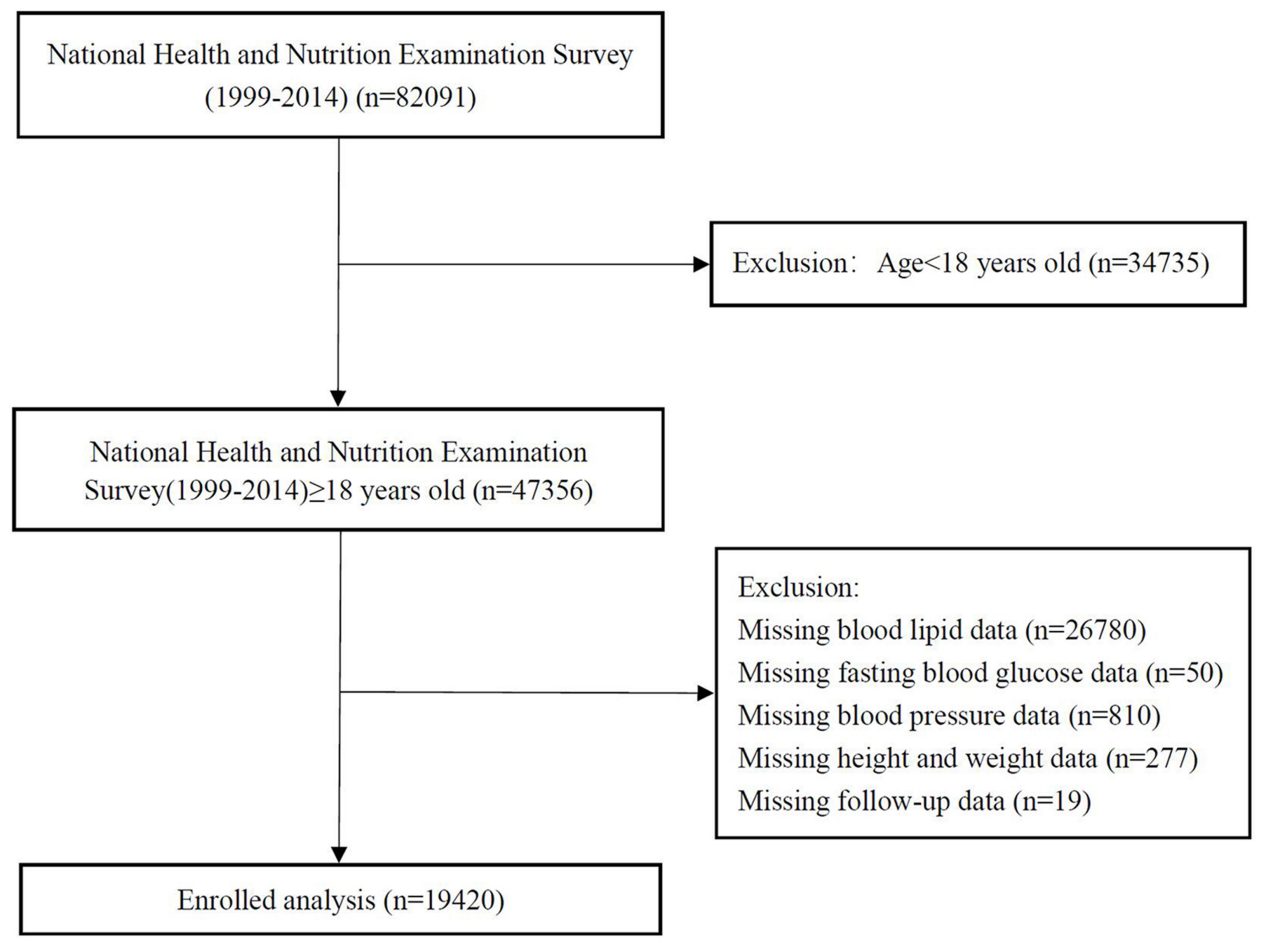
The research flow chart.

We analyzed data from the National Health and Nutrition Examination Survey (NHANES) 1999–2014. The data cut-off period was 31 December 2015. NHANES is a nationwide study to assess the health status of US citizens. The surveys were approved by the National Center for Health Statistics Research. Details of study implementation are available for online access (19). After excluding participants <18 years and those without complete medical records, 19,420 were included in the final analyses (Figure 1).

### Exposure

Blood specimen collection was conducted in the morning session after at least 9 h of overnight fasting. Hexokinase method was used to measure fasting blood glucose (20). The measurement of serum triglycerides (TG) and total cholesterol (TC) was performed with enzymatic assays. The level of high-density lipoprotein cholesterol (HDL-C) was determined by direct immunoassay or precipitation (21). If TG was ≤400 mg/dL, low-density lipoprotein cholesterol (LDL-C) was computed by the Friedewald formula (22). TyG index was defined as **TyG**=*Ln*[*fasting triglycerides* (*mg*/*dL*) × *fasting glucose* (*mg*/*dL*)/2] (9, 10).

### Covariates

With the use of standardized questionnaires, participants provided information on age, sex, race (classify as white or other), smoking (classify as yes or no), self-reported medical history, and medication use. Body mass index (BMI) was measured by anthropometric measurement, Modification of Diet in Renal Disease formula was used to compute the estimated glomerular filtration rate (eGFR) (23). CVD history was self-reported by the onset of coronary artery disease, angina, heart attack, or stroke. Blood pressure was also measured to identify participants with hypertension. The definition of hypertension was having systolic blood pressures (SBP) ≥140 mmHg, diastolic blood pressure (DBP) ≥90 mmHg, reported the usage of hypotensive drugs or history of hypertension (24). Participants were classified as having diabetes if fasting blood glucose ≥126 mg/dl, hemoglobin A1c ≥6.5%, reported the usage of hypoglycemic drugs or history of diabetes (25). Full procedures on data collection are available on the official website (26).

### Outcomes

In the present study, included outcomes were mortality cases due to all-cause and CVD. NHANES dataset was linked with the National Death Index (NDI) based on a probabilistic match to obtain mortality data. This match was performed by The National Center for Health Statistics with personal data, including birth date and social security number. Death certificate was used for confirmation whenever possible. These mortality files are available for online access (https://www.cdc.gov/nchs/data-linkage/mortality-public.htm). The definition of cardiovascular mortality in our study was adapted from the International Classification of Diseases, 10th Clinical Modification (ICD-10) System codes (I00–I09, I11, I13, I20–I51, I60–I69) (27).

### Statistical Analysis

Baseline characteristics of participants were classified by TyG index (<8, 8–9, 9–10, >10). For continuous variables, data were presented as average value with standard deviations (SD). For categorical variables, data were presented as numbers with percentages. Depending on the nature of data, Chi-square, ANOVA, or Kruskal-Wallis *H*-test were performed to detect subgroup differences. To examine the association of TyG index with mortality, three sets of Cox regression models were constructed. Model 1 only included TyG index. Model 2 was adjusted for age, sex, and race. On top of the variables in Model 2, SBP, eGFR, TC, HDL-C, CVD, diabetes, hypertension, hypotensive drugs, hypoglycemic drugs, lipid-lowering medication, and antiplatelet drugs were additionally adjusted in Model 3. A test of the trend across TyG index groups was also performed. The differential onset of all-cause mortality and cardiovascular death according to TyG index was tested by Kaplan–Meier survival analyses. The statistical significance in subgroup differences was evaluated by the log-rank test. Restricted cubic spline models were built to detect any non-linear relationship between TyG index and mortality. If non-linear relationships were identified, we used two-piecewise linear regression models to elucidate how the associations differed by the threshold point. The threshold value was estimated by trying all possible value and choosing the threshold point with highest likelihood. Logarithmic likelihood ratio test was employed to compare the differences in associations when using one-line linear regression models vs. two-piecewise linear regression models. We also evaluated how the results from regression analysis differed by age (≥65 years or <65 years), sex (male or female), race (white or other), diabetes (yes or no), hypertension (yes or no), and BMI (≥25 or <25 kg/m^2^). All analyses were performed with R version 3.6.3 (R Foundation for Statistical Computing, Vienna, Austria), with statistical significance being identified at the level of *P* < 0.05.

## Results

### Baseline Characteristics

Among 19,420 participants (48.9% men) in the present study, the average age was 47.1 (SD, 19.3) years. Across the average follow-up period of 98.2 (SD, 54.3) months, 2,238 (11.5%) death occurred and 445 (2.3%) died due to cardiovascular diseases. Table 1 has presented the remaining details of the baseline characteristics. The subgroup differences were statistically significant for all variables (all *P* < 0.001).

**Table 1:** Baseline characteristics according to triglyceride-glucose index.

	**Total**	**Triglyceride-glucose index**				***P*-value**
		**≤8**	**8–9**	**9–10**	**>10**	
Number	19,420	3,056	10,995	4,686	683	
Age, years	47.1 ± 19.3	36.5 ± 17.1	47.6 ± 19.5	52.6 ± 17.9	53.5 ± 15.5	<0.001
Sex, *n* (%)						<0.001
Male	9,494 (48.9)	1,235 (40.4)	5,301 (48.2)	2,538 (54.2)	420 (61.5)	
Female	9,926 (51.1)	1,821 (59.6)	5,694 (51.8)	2,148 (45.8)	263 (38.5)	
Race, *n* (%)						<0.001
Other	10,465 (53.9)	1,927 (63.1)	5,831 (53.0)	2,329 (49.7)	378 (55.3)	
White	8,955 (46.1)	1,129 (36.9)	5,164 (47.0)	2,357 (50.3)	305 (44.7)	
Smoking, *n* (%)						<0.001
No	9,672 (53.5)	1,668 (64.5)	5,551 (54.2)	2,158 (47.3)	295 (44.0)	
Yes	8,392 (46.5)	918 (35.5)	4,694 (45.8)	2,404 (52.7)	376 (56.0)	
BMI, kg/m^2^	28.4 ± 6.6	25.4 ± 5.8	28.1 ± 6.5	30.6 ± 6.2	31.2 ± 6.2	<0.001
SBP, mmHg	123.0 ± 19.1	115.9 ± 16.3	122.4 ± 18.9	127.8 ± 19.6	131.3 ± 20.5	<0.001
DBP, mmHg	68.8 ± 13.3	66.4 ± 11.8	68.6 ± 13.1	70.5 ± 13.9	71.1 ± 15.5	<0.001
eGFR, mg/min/1.73m^2^	91.1 ± 29.6	99.3 ± 26.8	90.6 ± 29.0	87.3 ± 31.5	88.8 ± 30.9	<0.001
TC, mg/dL	194.9 ± 42.9	169.3 ± 33.7	193.0 ± 38.7	210.4 ± 44.1	232.5 ± 61.1	<0.001
HDL-C, mg/dL	53.4 ± 15.8	62.2 ± 16.6	55.0 ± 15.1	46.1 ± 12.7	38.6 ± 11.3	<0.001
LDL-C, mg/dL	114.9 ± 35.9	96.8 ± 28.0	117.0 ± 34.3	122.1 ± 39.8	114.3 ± 40.5	<0.001
TG, mg/dL	135.9 ± 118.7	51.8 ± 11.6	104.9 ± 29.5	212.8 ± 66.4	484.1 ± 383.1	<0.001
Fasting blood glucose, mg/dL	105.6 ± 34.6	90.7 ± 9.6	99.2 ± 15.4	116.9 ± 37.4	197.8 ± 96.1	<0.001
Triglyceride-glucose index	8.66 ± 0.68	7.73 ± 0.23	8.51 ± 0.28	9.35 ± 0.26	10.49 ± 0.50	<0.001
**Comorbidities**, ***n*** **(%)**						
Hypertension						<0.001
No	11,327 (58.4)	2,398 (78.6)	6,574 (59.9)	2,111 (45.1)	244 (35.8)	
Yes	8,073 (41.6)	655 (21.5)	4,411 (40.2)	2,570 (54.9)	437 (64.2)	
Diabetes						<0.001
No	16,302 (84.0)	2,954 (96.7)	9,877 (89.9)	3,269 (69.8)	202 (29.6)	
Yes	3,112 (16.0)	101 (3.3)	1,115 (10.1)	1,415 (30.2)	481 (70.4)	
Cardiovascular disease						<0.001
No	16,204 (90.3)	2,407 (95.5)	9,286 (91.2)	3,954 (86.8)	557 (83.0)	
Yes	1,733 (9.7)	114 (4.5)	902 (8.9)	603 (13.2)	114 (17.0)	
**Treatment**, ***n*** **(%)**						
Hypotensive drugs						<0.001
No	14,479 (74.6)	2,723 (89.1)	8,369 (76.1)	2,997 (64.0)	390 (57.1)	
Yes	4,941 (25.4)	333 (10.9)	2,626 (23.9)	1,689 (36.0)	293 (42.9)	
Hypoglycemic drugs						<0.001
No	17,801 (91.7)	3,004 (98.3)	10,443 (95.0)	3,946 (84.2)	408 (59.7)	
Yes	1,619 (8.3)	52 (1.7)	552 (5.0)	740 (15.8)	275 (40.3)	
Lipid-lowering medication						<0.001
No	16,888 (87.0)	2,910 (95.2)	9,676 (88.0)	3,790 (80.9)	512 (75.0)	
Yes	2,532 (13.0)	146 (4.8)	1,319 (12.0)	896 (19.1)	171 (25.0)	
Antiplatelet drugs						<0.001
No	19,060 (98.2)	3,033 (99.3)	10,817 (98.4)	4,549 (97.1)	661 (96.8)	
Yes	360 (1.9)	23 (0.8)	178 (1.6)	137 (2.9)	22 (3.2)	
**Outcomes**, ***n*** **(%)**						
Cardiovascular disease mortality						<0.001
No	18,975 (97.7)	3,023 (98.9)	10,777 (98.0)	4,537 (96.8)	638 (93.4)	
Yes	445 (2.3)	33 (1.1)	218 (2.0)	149 (3.2)	45 (6.6)	
All-cause mortality						<0.001
No	17,182 (88.5)	2,906 (95.1)	9,770 (88.9)	3,986 (85.1)	520 (76.1)	
Yes	2,238 (11.5)	150 (4.9)	1,225 (11.1)	700 (14.9)	163 (23.9)	

*Values are presented as mean ± standardized differences or n (%)*.

### The Relationship of TyG Index With All-Cause and Cardiovascular Mortality

**Figure 2:**
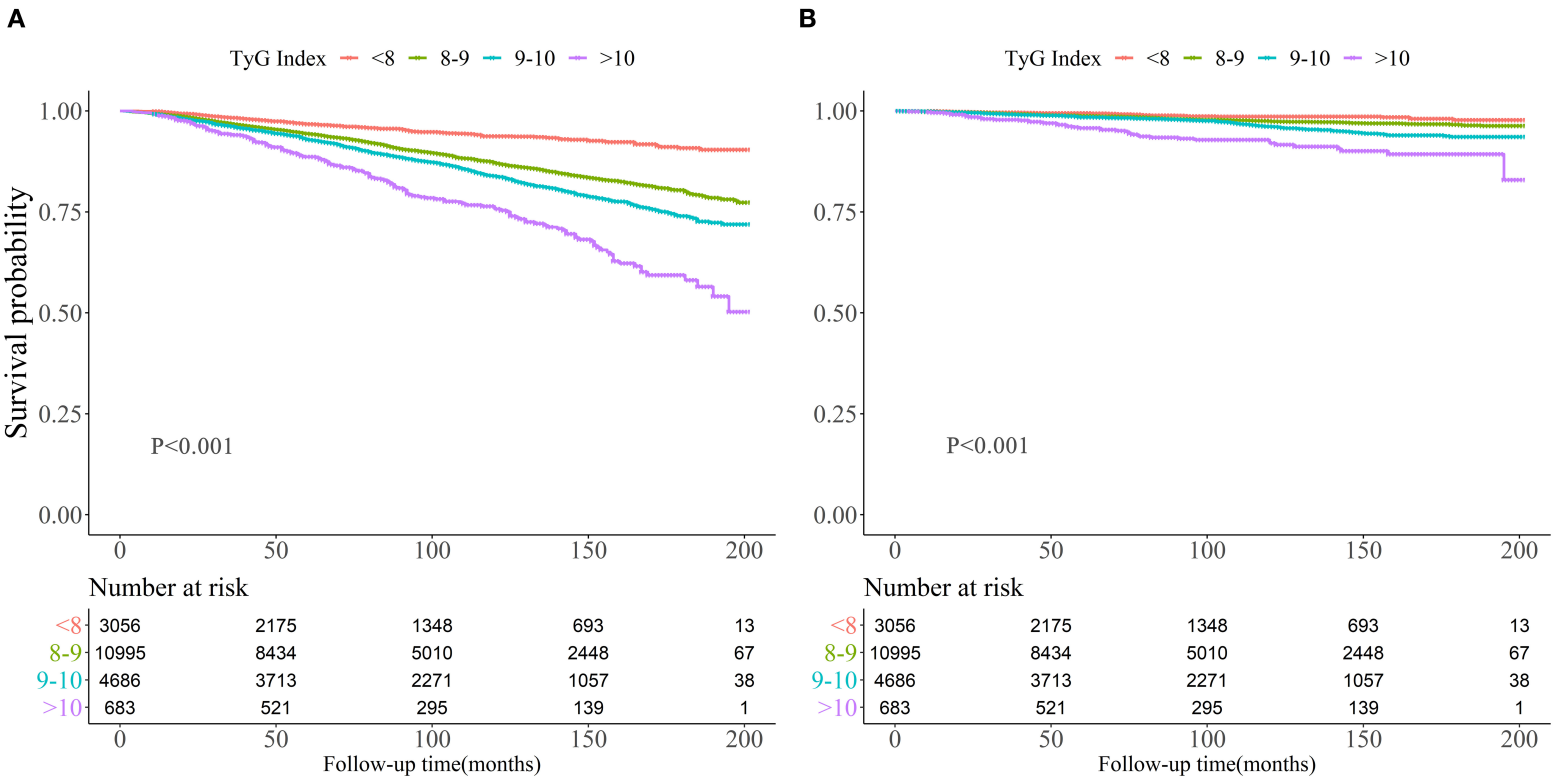
Kaplan-Meier survival curve for all-cause mortality **(A)** and cardiovascular mortality **(B)** by Triglyceride-glucose index.

Kaplan-Meier curves of the survival rate stratified by the TyG index were demonstrated in Figure 2. The cumulative incidence of death due to all-cause and CVD increased with TyG index (log-rank test, *P* < 0.001). Table 2 presents the results from Cox regression analysis. When treating TyG index as a continuous variable, a positive association with all-cause (HR, 1.10; 95% CI, 1.00–1.20) and cardiovascular mortality (HR, 1.29; 95% CI, 1.05–1.57) was found in Model 3. When treating TyG index as a categorical variable, compared with the lowest category (TyG index ≤8), participants with the highest TyG index (>10) had increased risks of all-cause (HR, 4.92; 95% CI, 3.94–6.14) and cardiovascular death (HR, 6.11; 95% CI, 3.90–9.58) in univariate analysis (Model 1). The association with all-cause mortality persisted in Model 3 (HR, 1.51; 95% CI, 1.15–1.98), but the positive effect sizes becoming non-significant with cardiovascular death (HR, 1.37; 95% CI, 0.78–2.42).

**Table 2:** Cox regression analysis of Triglyceride-glucose index with cause-specific mortality.

	**Number of events/1,000 person-years**	**Model 1**	**Model 2**	**Model 3**
**All-cause mortality**				
Triglyceride-glucose index (per 1 unit increment)	14.09	1.56 (1.48, 1.65) <0.001	1.14 (1.07, 1.22) <0.001	1.10 (1.00, 1.20) 0.047
Triglyceride-glucose index group				
≤8	6.17	1.0	1.0	1.0
8–9	13.64	2.21 (1.87, 2.62) <0.001	0.91 (0.77, 1.08) 0.29	0.93 (0.77, 1.11) 0.41
9–10	17.78	2.87 (2.41, 3.43) <0.001	0.92 (0.77, 1.10) 0.39	0.88 (0.72, 1.09) 0.24
>10	30.15	4.92 (3.94, 6.14) <0.001	1.71 (1.37, 2.13) <0.001	1.51 (1.15, 1.98) 0.003
*P* for trend		<0.001	<0.001	0.12
**Cardiovascular mortality**				
Triglyceride-glucose index (per 1 unit increment)	2.80	1.82 (1.62, 2.04) <0.001	1.44 (1.25, 1.66) <0.001	1.29 (1.05, 1.57) 0.014
Triglyceride-glucose index group				
≤8	1.36	1.0	1.0	1.0
8–9	2.43	1.78 (1.24, 2.57) 0.002	0.66 (0.46, 0.96) 0.028	0.63 (0.43, 0.93) 0.021
9–10	3.79	2.77 (1.90, 4.04) <0.001	0.81 (0.56, 1.19) 0.28	0.64 (0.41, 1.00) 0.048
>10	8.32	6.11 (3.90, 9.58) <0.001	2.02 (1.29, 3.17) 0.002	1.37 (0.78, 2.42) 0.27
*P* for trend		<0.001	<0.001	0.24

*Data are presented as hazard ratios, 95% confidence intervals, and P-value*.

*Model 1 adjusted for none*.

*Model 2 adjusted for age, sex, and race*.

*Model 3 adjusted for age, sex, race, smoking, BMI, SBP, eGFR, TC, HDL-C, comorbidities (cardiovascular disease, diabetes, and hypertension), and medicine use (hypotensive drugs, hypoglycemic drugs, lipid-lowering medication, and antiplatelet drugs)*.

### The Analyses of Non-linear Relationship

**Figure 3:**
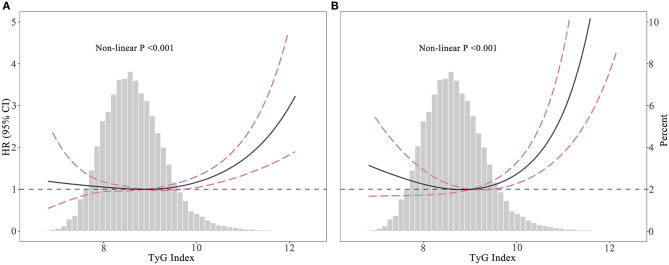
Hazard ratios for all-cause mortality **(A)** and cardiovascular mortality **(B)** according to Triglyceride-glucose index and the histogram of probability distribution was presented in the background (hazard ratios were calculated by Cox models after adjusting for age, sex, race, smoking, BMI, SBP, eGFR, TC, HDL-C, CVD, diabetes, hypertension, hypotensive drugs, hypoglycemic drugs, lipid-lowering medication, and antiplatelet drugs).

As shown in Figure 3, restricted cubic spline models suggested that the relationship between TyG index and mortality was non-linear (all non-linear *P* < 0.001). According to two-piecewise linear regression models (Table 3), the threshold value was 9.36 and 9.52 for all-cause and cardiovascular mortality, respectively. Below the threshold point, TyG index did not associate with all-cause mortality nor cardiovascular mortality. However, above the threshold point, the elevation in TyG index was associated with an increased risk of all-cause (HR, 1.50; 95% CI, 1.29–1.75) and cardiovascular (HR, 2.35; 95% CI, 1.73–3.19) mortality.

**Table 3:** The results of two-piecewise linear regression model between Triglyceride-glucose index and cause-specific mortality.

	**All-cause mortality**	**Cardiovascular mortality**
Threshold value	9.36	9.52
<threshold value	0.91 (0.81, 1.03) 0.14	0.90 (0.70, 1.16) 0.43
≥threshold value	1.50 (1.29, 1.75) <0.001	2.35 (1.73, 3.19) <0.001
*P* for log likelihood ratio test	<0.001	<0.001

*Data are presented as hazard ratios, 95% confidence intervals, and P-value*.

*The two-piecewise linear regression models were adjusted for age, sex, race, smoking, BMI, SBP, eGFR, TC, HDL-C, comorbidities (cardiovascular disease, diabetes, and hypertension), and medicine use (hypotensive drugs, hypoglycemic drugs, lipid-lowering medication, and antiplatelet drugs)*.

### Stratification Analysis

Table 4 showed the result of the stratification analysis. Above the threshold point, TyG index only associated with all-cause mortality among participants age ≥65 years (HR, 1.50; 95% CI, 1.29–1.75), those with hypertension (HR, 1.67; 95% CI, 1.41–1.99) or diabetes (HR, 1.55; 95% CI, 1.29–1.86). The association of TyG index with cardiovascular death was significant in male (HR, 2.59; 95% CI, 1.81–3.72), people suffering from hypertension (HR, 1.67; 95% CI, 1.41–1.99) or diabetes (HR, 1.55; 95% CI, 1.29–1.86).

**Table 4:** Stratification analysis of Triglyceride-glucose index with cause-specific mortality.

	**Number**	**All-cause mortality**		***P* for log likelihood ratio test**	**Cardiovascular disease mortality**		***P* for log likelihood ratio test**
**Threshold value, mmol/L**		**<9.36**	**≥9.36**		**<9.52**	**≥9.52**	
Age							
≥65	4,337	0.81 (0.70, 0.94) 0.005	1.47 (1.18, 1.82) <0.001	<0.001	0.82 (0.61, 1.10) 0.18	2.15 (1.34, 3.44) 0.001	0.003
<65	13,509	1.24 (1.01, 1.52) 0.037	1.15 (0.92, 1.45) 0.22	0.66	1.18 (0.72, 1.92) 0.51	1.76 (1.12, 2.79) 0.015	0.27
Sex							
Male	8,674	0.91 (0.78, 1.06) 0.23	1.52 (1.25, 1.86) <0.001	<0.001	0.99 (0.72, 1.36) 0.95	2.59 (1.81, 3.72) <0.001	<0.001
Female	9,172	0.92 (0.76, 1.11) 0.39	1.48 (1.16, 1.89) 0.002	0.006	0.82 (0.54, 1.24) 0.35	1.73 (0.94, 3.21) 0.080	0.080
Race							
Other	9,325	0.94 (0.78, 1.12) 0.48	1.37 (1.09, 1.71) 0.006	0.018	0.86 (0.59, 1.27) 0.46	2.91 (1.94, 4.36) <0.001	<0.001
White	8,521	0.91 (0.77, 1.07) 0.24	1.59 (1.28, 1.97) <0.001	<0.001	0.93 (0.66, 1.31) 0.68	1.77 (1.07, 2.93) 0.027	0.059
Hypertension							
No	9,908	1.05 (0.83, 1.35) 0.67	1.08 (0.76, 1.54) 0.68	0.93	1.54 (0.78, 3.05) 0.22	0.92 (0.34, 2.48) 0.88	0.40
Yes	7,938	0.85 (0.74, 0.98) 0.021	1.67 (1.41, 1.99) <0.001	<0.001	0.82 (0.63, 1.08) 0.16	2.79 (2.00, 3.88) <0.001	<0.001
Diabetes							
No	14,783	0.95 (0.82, 1.10) 0.49	1.37 (0.91, 2.08) 0.13	0.14	0.96 (0.69, 1.33) 0.80	0.39 (0.05, 2.85) 0.35	0.36
Yes	3,063	0.88 (0.70, 1.10) 0.25	1.55 (1.29, 1.86) <0.001	<0.001	1.04 (0.67, 1.61) 0.86	2.22 (1.55, 3.18) <0.001	0.018
BMI, kg/m^2^							
<25	5,516	0.88 (0.71, 1.08) 0.22	2.67 (1.87, 3.82) <0.001	<0.001	0.79 (0.50, 1.26) 0.32	5.30 (2.82, 9.96) <0.001	<0.001
≥25	12,330	0.93 (0.80, 1.08) 0.33	1.38 (1.16, 1.65) <0.001	0.002	0.93 (0.69, 1.27) 0.66	2.07 (1.43, 2.99) <0.001	0.003

*Data are presented as hazard ratios, 95% confidence intervals, and P-value*.

*When analyzing a stratification variable, age, sex, race, smoking, BMI, SBP, eGFR, TC, HDL-C, comorbidities (cardiovascular disease, diabetes, and hypertension), and medicine use (hypotensive drugs, hypoglycemic drugs, lipid-lowering medication, and antiplatelet drugs) were all adjusted except the variable itself*.

## Discussion

Our analysis has demonstrated the non-linear relationship of TyG index with mortality due to all-cause and CVD among US population. We also identified the threshold points where the association of TyG index with mortality was further increased.

Patients with IR were often accompanied by lipid and glucose metabolism disorders (16). The phenomenon of lipid-induced insulin resistance can be explained by glucose-fatty acid cycle hypothesis (28). Therefore, TyG index can serve as a practical alternative of IR measurement (29). When compared to HOMA-IR, Lee et al. found that TyG index is a better indicator of arterial stiffness in Korean adults (18). Chiu et al. and Zhao et al. found that TyG index was associated with macrovascular and microvascular damage (30, 31). TyG index was also found to associate with CVD incidence (14, 32), hypertension, diabetes, as well as metabolic syndrome (11, 12, 33). Our analyses demonstrated the threshold values to detect mortality risk using TyG index. In a study of 2,531 subjects, Wang et al. found the threshold value of TyG index to predict adverse cardiovascular events was 9.32 among patients with diabetes and acute coronary syndrome (34), which was similar to the threshold values we have found. However, not all studies have found significant associations. Laura et al. failed to detect any significant associations between the TyG index and CVD for people with diabetes or hypertension (35). Cho et al. reported that TyG index had no significant relationship with coronary artery disease among diabetic participants after adjusting for covariates (36).

The detail in the mechanism accounting for the relationship between TyG index and death is still under investigation. Several speculations have been summarized as follows. First, TyG index had a close relationship with a broad range of adverse health conditions including obesity, diabetes, CVD, higher blood pressure, lower eGFR, and HDL-C levels (11, 12, 37). Elevated TyG index may reflect the adverse effects of impaired cardiometabolic health. In our stratified analysis, the positive association of the TyG index with mortality was more profound among older participants and people with pre-existing diseases. Second, endothelial dysfunction may have a role in the association of TyG index with mortality. Compared with other IR indicators, TyG index mainly quantifies IR in muscle and is a better indicator for peripheral IR. Therefore, TyG index probably relates to the level of endothelial dysfunction, oxidative stress and inflammatory response (1, 3, 38). Positive associations of the TyG index with white blood cells count and high-sensitivity C-reactive protein levels were also revealed in previous studies (16, 17). Inflammation may impair vascular endothelium. That can lead to the leakage of blood contents into the perivascular spaces and further cause vascular damage (39). Third, insulin may cause lipohyalinosis through the elevation of sympathetic activity, which may lead to diffuse hypoperfusion or blocks small perforating arterioles (37, 39). IR can also promote atherogenesis and advanced plaque progression (37). Fourth, TyG index predicts the incidence of prediabetes (40), while meta-analyses have confirmed that prediabetes was positively associated with all-cause and CVD death in general population (41, 42). Nevertheless, the precise role of the TyG index in mortality remains to be investigated.

Despite the important findings being mentioned, some limitations of this study need to be recognized. First, insulin clamp was not available in this study, and thus we could not use the most accurate methods to evaluate IR. Second, the present study does not imply causality due to the cross-sectional nature. Lastly, the finding was mainly applicable in the United States and may not be directly extrapolated to other regions and ethnic groups.

## Conclusions

There is a non-linear relationship of TyG index with mortality due to all-cause and CVD among US population. Whether the improvement of TyG index may reduce mortality risk in long run need to be further evaluated by intervention studies.

## Data Availability Statement

Publicly available datasets were analyzed in this study. This data can be found at: https://www.cdc.gov/nchs/nhanes/index.htm.

## Ethics Statement

The studies involving human participants were reviewed and approved by the Institutional Review Board of the Centers for Disease Control and Prevention (Protocol 98–12, 2005–06, and 2011–17). The patients/participants provided their written informed consent to participate in this study.

## Author Contributions

Y-qH, G-dH, KL, Y-qF, and X-cL: conceptualization and methodology. Y-qH and X-cL: formal analysis. Y-qH and Y-qF: supervision and validation. X-cL and KL: writing and revision. All authors: contributed to the article and approved the submitted version.

## Conflict of Interest

The authors declare that the research was conducted in the absence of any commercial or financial relationships that could be construed as a potential conflict of interest.
